# Motor learning is modulated by dopamine availability in the sensorimotor putamen

**DOI:** 10.1093/braincomms/fcae409

**Published:** 2024-11-13

**Authors:** Christoph Muehlberg, Sophia Goerg, Michael Rullmann, Swen Hesse, Osama Sabri, Max Wawrzyniak, Joseph Classen, Christopher Fricke, Jost-Julian Rumpf

**Affiliations:** Department of Neurology, Leipzig University Medical Center, 04103 Leipzig, Germany; Department of Neurology, Leipzig University Medical Center, 04103 Leipzig, Germany; Department of Nuclear Medicine, Leipzig University Medical Center, 04103 Leipzig, Germany; Department of Nuclear Medicine, Leipzig University Medical Center, 04103 Leipzig, Germany; Department of Nuclear Medicine, Leipzig University Medical Center, 04103 Leipzig, Germany; Department of Neurology, Leipzig University Medical Center, 04103 Leipzig, Germany; Department of Neurology, Leipzig University Medical Center, 04103 Leipzig, Germany; Department of Neurology, Leipzig University Medical Center, 04103 Leipzig, Germany; Department of Neurology, Leipzig University Medical Center, 04103 Leipzig, Germany

**Keywords:** motor learning, dopamine transporter, dopamine, striatum, Parkinson's disease

## Abstract

Successful motor skill acquisition requires the dynamic interaction of multiple brain regions, with the striatum playing a critical role in this network. Animal studies suggest that dopaminergic mechanisms are involved in the regulation of motor learning–associated striatal plasticity. In humans, however, the contribution of nigrostriatal dopaminergic transmission to motor learning remains elusive beyond its well-characterized role in initiation and fluent execution of movements. In this prospective observational study, we investigated motor sequence learning in individuals who had undergone ^123^I-N-ω-fluoropropyl-2β-carbomethoxy-3β-(4-iodophenyl)nortropane single-photon emission computed tomography for the differential diagnosis of Parkinson's disease (*n* = 41) and age-matched healthy controls (*n* = 20). We found that striatal dopamine transporter depletion exhibited distinct spatial patterns that were associated with impairments in motor sequence learning and the manifestation of Parkinsonian motor symptoms, respectively. Specifically, significant associations between striatal dopamine transporter depletion and impairments in motor sequence learning were confined to posterior putaminal regions, whereas significant associations of striatal dopamine transporter depletion with Parkinsonian motor symptom severity showed a widespread spatial pattern across the entire striatal volume with an anterior maximum. Normative functional connectivity analysis revealed that both behavioural domains shared largely overlapping connectivity patterns with the basal ganglia and supplementary motor area. However, apart from connectivity with more posterior parts of the supplementary motor area, significant functional connectivity with primary motor cortical areas was only present for striatal dopamine transporter availability–related modulation of online motor learning. Our findings indicate that striatal dopaminergic signalling plays a specific role in motor sequence learning beyond its influence on mere motor execution, implicating learning-related sensorimotor striatum recruitment and cortico-striatal plasticity as dopamine-dependent mechanisms.

## Teaser

Muehlberg *et al*. show that striatal dopaminergic signalling is crucial for motor learning, beyond its role in motor execution alone. Motor learning impairments were notably linked to dopamine depletion in posterior putaminal regions connected to the primary motor cortex, indicating that cortico-striatal plasticity involved in motor learning is dopamine dependent.

## Graphical Abstract

**Graphical Abstract:**
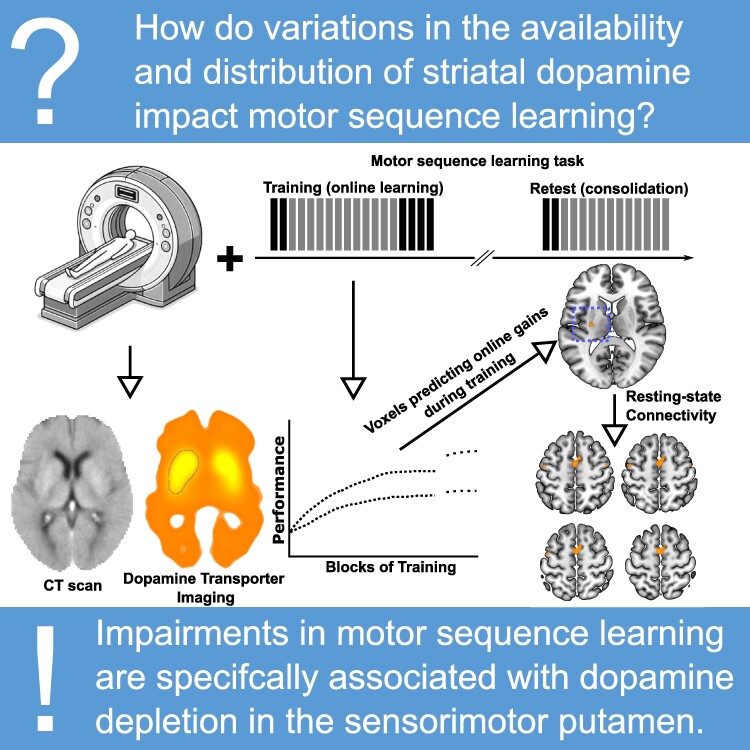

## Introduction

The ability to acquire, to perfect and to maintain motor skills is a central aspect of human life and critical to maintaining functional autonomy throughout the lifespan. Traditionally, motor learning is categorized into different phases: online and offline learning, which are sustained by distinct yet interconnected and complementary neural mechanisms.^1-3^ Online motor learning pertains to enhancements in motor performance observed during active practice, while offline motor learning encompasses the consolidation of practice-induced motor skill formation that occurs in the absence of further active practice, typically across periods of rest or sleep.^2-7^ Moreover, while the initial online learning phase is characterized by rapid performance improvements driven by the integration of sensory inputs, subsequent online and offline learning are marked by slower and more incremental performance changes.^1^ A widely employed and ecologically valid paradigm for investigating the behavioural aspects and neural mechanisms of motor skill acquisition is motor sequence learning, which entails the integration of a series of individual movements into a cohesive and fluently executed unit through repeated practice.^8,9^ Motor sequence learning underpins a multitude of daily activities, including typing, smartphone usage and playing a musical instrument. This process involves intricate cortical and sub-cortical interactions, where the dynamic integration of associative and sensorimotor striatal pathways plays a pivotal role in acquiring motor skills across early online, later online and offline learning phases.^10-13^

Due to its reliance on repeated practice, acquisition and maintenance of motor skills may be particularly compromised in contexts marked by impaired movement execution.^14^ In Parkinson’s disease, initiation and fluent execution of voluntary movements are progressively impaired over the course of the disease by disease-defining motor symptoms such as bradykinesia and rigidity. The cardinal Parkinsonian motor symptoms result from the gradual degeneration of dopaminergic projections originating from the substantia nigra pars compacta to the dorsal striatum (i.e. caudate nucleus and putamen).^15-18^ However, while deficits in striatal dopamine levels are causally associated with the progressive impairment of movement initiation and execution characteristic of Parkinson’s disease, it remains uncertain whether and to what extent striatal dopamine depletion influences motor skill acquisition independently of its impact on motor execution. Motor learning heavily relies on the dynamic activation of striatal pathways,^10,13,19,20^ and evidence from animal research indicates that cellular plasticity associated with motor learning in the sensorimotor striatum depends on dopamine.^21^ Consequently, the depletion of striatal dopamine may potentially hinder the acquisition of new motor skills. However, investigations into motor sequence learning in Parkinson’s disease provided largely inconsistent results with respect to the question whether disease-related striatal dopamine depletion correlates with deficits in motor learning.^22-32^ In addition, although pharmacological dopamine replacement therapy has been shown to improve motor learning in the elderly, possibly by counteracting age-related striatal dopamine depletion,^33-35^ the limited evidence regarding the beneficial effects of dopamine replacement on motor learning in Parkinson’s disease^36-40^ remains inconsistent.

In the current study, we examined the impact of varying levels and spatial distribution of striatal dopamine transporter availability on the acquisition of a dexterous sequential motor skill. Striatal dopamine availability was assessed by applying ^123^I-N-ω-fluoropropyl-2β-carbomethoxy-3β-(4-iodophenyl)nortropane single-photon emission computed tomography (^123^I-FP-CIT-SPECT, DaT-SPECT) for the differential diagnosis of Parkinson’s disease. Employing a motor sequence learning task in conjunction with voxel-wise analysis of DaT-SPECT data and normative resting-state connectivity, our investigation seeks to shed light on the distinct role of nigro-striatal dopaminergic function in motor learning apart from its role in regulating overall motor execution.

## Materials and methods

The study protocol was approved by the local Ethics Committee (registration code: 306/21-ek) and was registered in the German Clinical Trials Register (24 August 2021; DRKS00026167).

### Participants

Sixty-one individuals (mean age, 64.6 ± 8.0 years; 24 female) were recruited for the study between August 2021 and May 2023. Forty-one of the participants were recruited from the Department of Nuclear Medicine at the Leipzig University Medical Centre after having received DaT-SPECT for differential diagnosis of suspected Parkinson’s disease (DaT-SPECT group: mean age, 64.2 ± 8.7; 14 female). In addition, 20 age- and sex-matched healthy controls were recruited via local announcements (control group: mean age, 65.3 ± 6.6; 10 female). All participants were naive to the applied motor sequence learning task. Exclusion criteria were current or previous alcohol/illicit drug abuse, acute psychosis, frequent falls, levodopa equivalence dose of > 600 mg/d, relevant cognitive impairment defined as a score below 18 in the Montreal cognitive assessment,^41^ and severe internal medicine or orthopaedic conditions that could have interfered with task execution. Participants were screened for symptoms of depression using the beck depression inventory.^42^

At the time of the experiment, 10 out of the 41 participants in the DaT-SPECT group had already started dopaminergic medication because of suspected Parkinson’s disease (mean levodopa equivalent dose: 170.7 ± 105.3 mg; range, 50–300 mg per day). Dopaminergic medication was paused at least 14 h (24 h in three participants on dopamine agonists) prior to the initial motor training session so that all participants were off dopaminergic medication during the motor learning experiment. Parkinsonian motor symptoms were assessed by applying the motor section of the Movement Disorder Society-Unified Parkinson's Disease Rating Scale (MDS-UPDRS part III^43^). Additionally, we calculated an upper extremity sub-score (UES) from the MDS-UPDRS III for each participant, focusing on the hand used to perform the motor learning task. This score was defined as the sum of the items most relevant to affect task execution: ‘rigidity’, ‘finger tapping’, ‘hand movements’ and ‘pronation-supination movements’, with a total possible score ranging from 0 to 16 points, where higher values indicate greater symptom severity. Subjective impairment due to Parkinsonian motor symptoms was assessed using the MDS-UPDRS part II.^43^ Data from three participants were excluded from the final analysis: one did not refrain from repracticing the motor sequence learning task during the 6-h consolidation interval between the training and delayed retest sessions (task instructions see below), one had a hydrocephalic configuration on the brain CT scan that prevented proper normalization of the imaging data and one was excluded due to poor quality of the DaT-SPECT images. Therefore, 38 participants finally formed the DaT-SPECT group (please see Table 1 for demographic and clinical details). According to the Edinburgh Handedness Inventory,^44^ 34 participants in the DaT-SPECT group were right-handed (89.5%), 3 were ambidexterous (7.9%), 1 one (2.6%) was left-handed. In the control group, 17 participants were right-handed (85%), 2 were ambidexterous (10%) and 1 was left-handed (5%).

**Table 1:** DaT-SPECT group characteristics

*n*	Age	Sex	U total	UES	SBR CN	SBR *P*	*z-*value CN	*z*-value *P*	LEDD	Clinical diagnosis
1	47	F	24	10	1.88	1.09	−4.22	−5.45	0	PD
2	62	M	30	11	2.1	1.15	−2.98	−4.87	52	PD
3	69	M	3	0	2.72	2.61	−1.32	−0.77	0	RBD
4	64	F	23	6	2.74	2.93	−1.27	−0.24	0	PD
5	57	F	27	11	1.58	1.13	−4.52	−5.18	150	PD
6	70	M	38	7	3.05	2.3	−0.45	−1.52	300	PD
7	72	M	12	2	2.3	2.11	−1.78	−1.6	0	ET
8	56	M	10	1	2.09	1.81	−2.46	−2.64	0	ET
9	75	F	20	8	1.22	0.81	−3.63	−4.1	250	PD
10	64	M	27	9	1.29	0.78	−3.97	−4.56	0	PD
11	53	M	16	5	2.08	1.91	−2.72	−2.54	0	ET
12	61	M	17	5	1.35	0.8	−3.74	−4.49	0	PD
13	73	F	4	0	2.69	2.74	−0.86	−0.36	50	ET
14	46	M	6	0	2.44	2.02	−2.06	−2.5	0	ET
15	60	M	36	11	1.4	1.3	−3.88	−3.63	100	PD
16	63	M	12	4	1.99	1.7	−2.48	−2.67	0	PD
17	54	M	35	9	1.71	1.52	−3.41	−3.32	0	PD
18	74	M	30	6	1.55	1.22	−3.19	−3.37	300	PD
19	58	M	12	3	1.32	0.91	−4.07	−4.47	105	PD
20	75	M	32	8	1.51	0.89	−3.07	−3.94	0	PD
21	68	F	37	9	1.78	1.02	−2.9	−3.95	0	PD
22	52	F	17	1	3.23	2.83	−0.52	−0.68	0	ET
23	65	M	18	6	1.44	0.86	−3.64	−4.37	0	PD
24	72	F	8	2	3.05	3.04	−0.32	−0.32	0	ET
25	72	M	36	10	1.21	0.83	−3.92	−4.24	300	PD
26	68	M	15	6	1.52	1.22	−3.41	−3.55	0	PD
27	72	F	9	2	2.25	2.33	−1.88	−1.14	0	ET
28	54	M	5	1	2.52	2.52	−1.84	−1.27	0	ET
29	75	M	28	5	2.42	1.94	−1.32	−1.88	0	PD
30	70	M	9	1	2.15	1.96	−1.99	−1.97	0	ET
31	67	F	9	1	2.91	2.73	−0.73	−0.46	0	ET
32	71	F	7	1	2.88	2.8	−0.67	−0.2	0	ET
33	69	F	32	7	1.96	1.3	−2.39	−3.33	100	PD
34	67	M	13	5	1.44	1.06	−3.42	−3.82	0	PD
35	70	F	9	1	2.16	1.95	−1.96	−1.99	0	ET
36	55	M	4	0	2.44	2.15	−1.97	−1.99	0	ET
37	68	F	4	0	2.23	1.88	−1.88	−2.19	0	ET
38	72	M	6	0	2.41	2.25	−1.57	−1.3	0	ET

ET, essential tremor; F, female; LEDD, levodopa equivalent dose (mg); M, male; PD, Parkinson’s disease; RBD, Rapid eye movement sleep behaviour disorder; SBR P, specific binding ratio (Hermes BRASS) of putamen; U total, MDS-UPDRS III total score; UES, MDS-UPDRS-III upper extremity sub-score of the task performing hand including items for rigidity and bradykinesia (range, 0–16). SBR CN, specific binding ratio (Hermes BRASS) of caudate nucleus; *z*-value CN, age-adjusted *z*-value for caudate nucleus; *z*-value *P*, age-adjusted *z*-value for putamen (values presented for the striatum contralateral to the hand used for task performance.

### DaT-SPECT imaging and data processing

SPECT was performed 3 h after intravenous injection of about 185 MBq [^123^I]FP-CIT (DaTSCAN^TM^, GE HealthCare, Braunschweig, Germany) using a dual head Symbia Intevo Bold camera system (Siemens Healthineers, Forchheim, Germany) equipped with low-energy, high-resolution collimators. The acquisition parameters consisted of a rotational radius of 14.0 cm, a 20% energy window centred on 159 keV, 60 projection angles over 180° (25–30 s per projection depending on the count rate), a 128 × 128 matrix with a zoom factor of 1.23. To protect against irradiation of the thyroid, the subjects were pretreated with perchlorate 60 min before intravenous radiotracer injection. Images were reconstructed by iterative reconstruction and corrected for attenuation according to Chang (µ = 0.12 cm^−1^) and scatter as it is implemented in Hermes Hybrid Siemens Recon Neuro (Hermes Medical Solutions, Stockholm, Sweden) and analysed using the Hermes Brain Registration and Analysis Software Suite (BRASS, Hermes Medical Solutions, Stockholm, Sweden) to obtain specific binding ratios (SBR) and *z*-scores for the regions-of-interest, i.e. the entire striatum, the head of the caudate, and the putamen.^45^ Higher SBR values reflect a greater density of dopamine transporters in the region of interest (normalized to an occipital reference region), while lower SBR values indicate a reduction in dopamine transporter density. The SBR *z*-score, calculated by the Hermes BRASS tool, represents the number of SDs which the patient’s SBR deviates from the mean SBR of an age-matched healthy control group. A *z*-score of lower than −2.5 is considered suggestive of a neurodegenerative Parkinsonian syndrome.

### Experimental procedure

**Figure 1:**
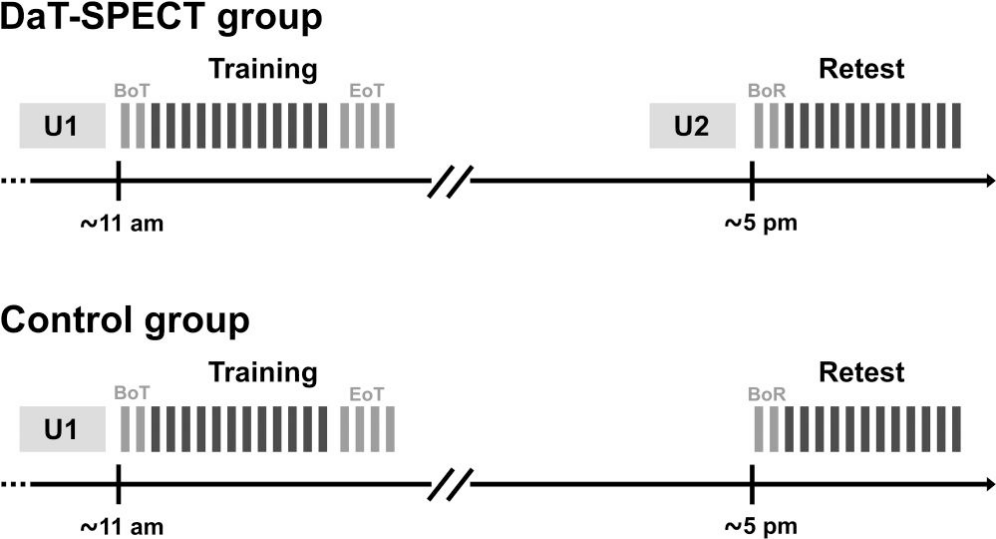
**Experimental design**. After assessment of Parkinsonian motor symptoms according to the MDS-UPDRS III (U1), all participants performed a motor sequence training session (training), which consisted of 18 blocks of task practice. The first two blocks of the training session were defined as the beginning of training baseline (BoT) and blocks 15 through 18 were defined as the end of training baseline (EoT). After an interval of 6 h, all participants completed a retest session (retest), which consisted of 14 blocks of the motor sequence learning task. The first two blocks of retest were defined as the beginning of retest (BoR). Participants who underwent dopamine transporter single-photon emission computed tomography (DaT-SPECT group) received an additional assessment of Parkinsonian motor symptoms before the retest session (U2), to exclude fluctuations of Parkinsonian motor symptoms as a potential source of task performance differences.

The motor learning experiment consisted of a motor sequence training session in the morning (training) and a delayed retest of task performance after an interval of 6 h in the afternoon (retest) of the same day to assess offline consolidation of training-induced task performance improvements (Fig. 1).

### Motor sequence learning task

Motor learning was assessed with a modified version of the explicit sequential finger tapping task initially introduced by Karni *et al*.^8^ Participants were asked to practice a five-item sequence of finger taps on a customized gaming keyboard (sequence: 4-1-3-2-4, where 1 = index finger, 2 = middle finger, 3 = ring finger and 4 = little finger). Participants in the DaT-SPECT group performed the task with the hand that was most affected by motor symptoms (bradykinesia and rigidity). In case of absence of Parkinsonian motor symptoms or symmetrical expression of symptoms, the hand contralateral to the side with the numerically lower putaminal SBR value was chosen to perform the task. In the control group, the hand to perform the task was chosen so that it approximately matched the distribution of task performance with the right or left hand in the DaT-SPECT group. Following this procedure, 16 out of 38 participants in the DaT-SPECT group and 7 out of 20 healthy controls completed the motor learning task with their left hand. This resulted in 21 of 38 participants (61.8%) in the DaT-SPECT group using their dominant hand, while 13 participants (38.2%) used their non-dominant hand. Among the three ambidexterous individuals in the DaT-SEPCT group, two used their left hand and one used the right hand. In the control group, 12 of 17 right-handed participants (70.6%) performed the task with their dominant hand, and five participants (29.4%) used their non-dominant hand. The two ambidexterous control participants each used a different hand, with one using the left hand and the other using the right hand. Prior to the onset of the actual training session, participants were asked to repeat the finger-tapping sequence slowly and accurately until they managed to reproduce it three times in a row without producing erroneous taps to verify explicit knowledge of the sequence. The training session comprised 18 blocks that were separated by 25-s rest blocks. Between blocks 14 and 15, the rest interval was extended to a break of 2 min to allow for relaxing the hand prior to performance of the last four blocks of the training session, which served as the end of training (EoT) baseline against which online and offline performance changes were assessed (see below). A retest session was conducted after 6 h on the same day and consisted of 14 blocks of task performance. An active practice block was indicated by a green fixation cross displayed on a computer screen in front of the participants, while a red fixation cross indicated a rest block. Participants were instructed to perform the sequence as fast as possible while making as few errors as possible. Unknown to participants, each active practice block was terminated after 60 button presses, resulting in a maximum of 12 correct sequences per block. This design ensures that all participants perform the same number of finger movements independent of the speed of task performance. After termination of the training session, participants were instructed to refrain from mentally or physically practicing the task during the consolidation period between the training and retest sessions.

### Statistical analysis of behavioural data

We used customized Matlab scripts (Mathworks, Natick, MA, USA) to extract the speed and accuracy of sequence execution from the recorded timing of each keystroke. Task performance in terms of speed was defined as the average time (seconds) required to execute a correct sequence within a given block (correct sequence duration). Accuracy was defined as the ratio of the number of correctly performed sequences divided by the maximum number of correct sequences per block (i.e. 12; ACC). To account for potential inter-individual differences in the strategy to improve task performance (e.g. focusing on speed performance at the expense of accuracy, or vice versa), we calculated a performance index (PI)^28,46^ that combines speed performance and accuracy of task execution according to the following formula:


(1) $$\begin{array}{rl} & \mathrm{PI}(x)\;=\;100\;\times \;e^{-\mathrm{CSD}(x)}\;\times \;e^{\mathrm{ACC}(x)-1},\\ & \;\mathrm{where}\;\;x\;=\;\mathrm{block}\;\mathrm{number} \end{array}$$

Effects of repeated task execution on task performance across the initial training session (online learning) and the retest session were assessed by applying separate repeated measures analyses of variance (rmANOVA). In case rmANOVA revealed a significant interaction of within- and between-subject factors, we used independent-sample *t*-tests for follow-up analysis. Online learning across the initial training session was operationalized as the difference between the EoT baseline (mean PI of the last four blocks of the training session) and the average task performance at the beginning of the training session (BoT, i.e. mean PI of the first two blocks of the training session). Changes in performance that were generated offline (i.e. consolidation) were assessed as the difference between the task performance at the beginning of the retest session (BoR, mean PI of the first two blocks of the retest) and the average PI across the EoT baseline (mean PI across the last four blocks of the training session). Two participants failed to produce a correct sequence in either one of the initial two blocks of the retest session. In this case, the missing PI value was imputed by the value of the other of the two blocks at the onset of retest. Demographic data were checked for normal distribution using the Kolmogorov–Smirnov test. In case of not normally distributed data, we applied Mann–Whitney U*-*test for between-group comparisons. Correlations of task execution relevant Parkinsonian motor symptoms (as assessed by UES) were computed with DaT-SPECT SBR values, online task performance changes and offline consolidation.

Statistical analyses were performed with SPSS® 29 (IBM, Armonk, NY, USA) and MATLAB (Mathworks, Natick, MA, USA). The alpha level was set to *P* < 0.05 for all statistical tests. The rmANOVAs were checked for violation of sphericity, and the degrees of freedom and *P*-values were corrected accordingly with Huyn–Feldt correction, if necessary.

### Voxelwise DaT-SPECT analysis

For each participant in the DaT-SPECT group, we had access to both a brain CT scan and the individual DaT-SPECT scans. CT scans were utilized to enable normalization of the DaT-SPECT data, allowing voxelwise analysis of the dopaminergic deficit on a group level and the associations to behavioural assessments. The voxelwise approach allows uncovering whether sub-regions within the striatum feature significant associations with different behavioural parameters, which is not possible using the global SBR values.

**Figure 2:**
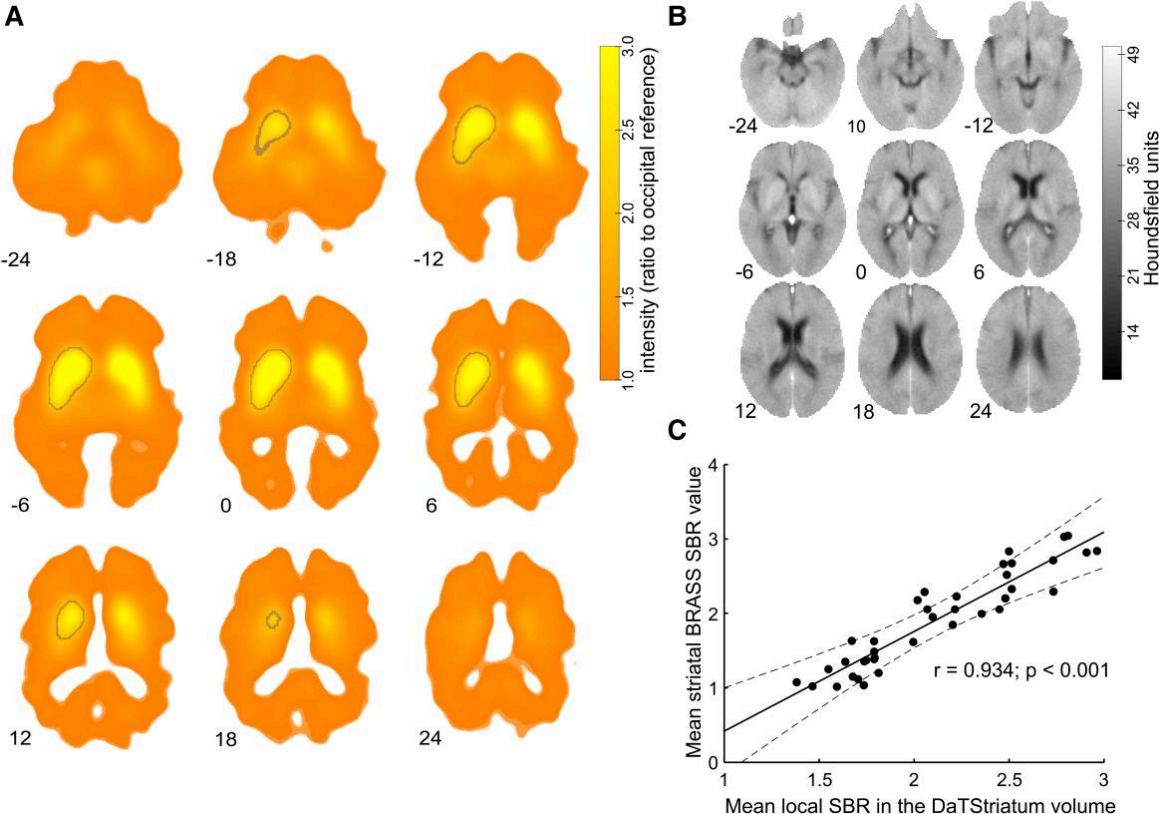
**DaT-SPECT and brain computer tomography scan normalization**. (**A**) Average normalized and standardized (occipital reference) and—if appropriate—mirrored DaT-SPECT scans after individual coregistration to CT scans (*n* = 38 participants). The volume of interest (standardized DaT-SPECT intensity > 2.0 relative to an occipital reference, termed ‘DaTStriatum’) is outlined by a grey line. Scans were mirrored right to left when participants trained with their left hand so that the DaTStriatum volume of interest was always on the contralateral left side for group level analysis. (**B**) Average CT scans of the participants after normalization to MNI space, same slices as in Fig. 2A. (**C**) Scatter plot of mean standardized DaT-SPECT intensity from the DaTStriatum volume (‘local SBR’) and mean (caudate nucleus and putamen) striatal SBR value according to the Hermes BRASS algorithm (*n* = 38; *r* = 0.934; *P* < 0.001).

First, each DaT-SPECT scan was aligned with the individual brain CT scan through an affine transformation, followed by manual refinement using 3DSlicer (version 4.11, slicer.org^47^). Subsequently, all scans were transformed into Montreal Neurological Institute (MNI) space using the ‘Clinical toolbox’^48^ and SPM12 for Matlab (Fig. 2A). The toolboxes allowed for normalization of CT scans and the resulting individual transformation matrices were used to transform the previously co-registered DaT-SPECT volumes (Fig. 2B). To ensure consistency in tracer binding across scans, the DaT-SPECT data were rescaled. This rescaling involved dividing the value at each voxel by the average of the voxels corresponding to Brodmann areas 17, 18 and 19 (occipital cortex) and yielded the ‘local SBR’. Then, a mask was generated by averaging all normalized and rescaled DaT-SPECT data, excluding values below 2.0 times the occipital reference (Fig. 2A, area encircled by grey line). This process effectively isolated the striatal volumes within the individual DaT-SPECT data. The resulting volume is from now on termed ‘DaTStriatum’. Given that the motor learning task was performed with either the left or right hand only, we focused our analysis on the contralateral striatum. To enable statistical analysis, scans were mirrored (right to left) for subjects who practiced the motor task with their left hand. DaT-SPECT data from the DaTStriatum contralateral to the hand used for task execution were then used to further investigate associations between online and offline motor sequence learning and task execution-relevant Parkinsonian motor symptoms (i.e. UES values) with the spatial distribution of ^123^I-FP-CIT binding. The mean of the local SBR across the individual DaTStriatum volume was calculated and correlated with the SBR values used for standard clinical purposes as quantified with Hermes BRASS^49^ to gauge the validity of this preprocessing, which yielded a very high positive correlation (*r* = 0.934; *P* < 0.001; Fig. 2C). Additionally, we calculated correlations between the MNI coordinates of the maximum intensity voxel as well as the centre of gravity of the local SBR within the DaTStriatum volume and behavioural metrics. This analysis was conducted based on the expectation that dopamine depletion is not uniformly distributed across the DaTStriatum volume, with a more pronounced depletion in the lateral putamen.^50^ Consequently, this would lead to a shift of the higher intensity voxels towards more medial coordinates. We then employed voxelwise regression as implemented in SPM12 to evaluate linear dependencies of the local SBR within the DaTStriatum volume and (i) the magnitude of task execution–relevant Parkinsonian motor symptoms (UES), as well as (ii) online and (iii) offline motor sequence learning. This yielded the amount to which each local SBR voxel in the DaTStriatum volume was associated with each of the behavioural metrics. For statistical analysis, significance of these associations was assessed at cluster level [significance defined for FWE-corrected *P*-values <0.05 using *P*(uncorrected) < 0.001 as the cluster defining threshold]. Peak voxels of significant clusters (as centre of a sphere with a radius of 5 mm) were used as seeds to evaluate the connectivity of the different striatal areas found to be associated with the behavioural metrics in a second level analysis involving normative resting-state fMRI data.

To achieve this, we employed publicly available resting-state functional MRI data obtained from a cohort of 100 unrelated, healthy young individuals included in the Human Connectome Project.^51^ Analysis was conducted as previously described in study by Wawrzyniak *et al*.,^52^ Klingbeil *et al*.^53^ and Muehlberg *et al*.^54^ Each fMRI dataset comprised two resting-state sessions (right-to-left and left-to-right phase encoding), each acquired during a 15-min scan using a gradient-echo planar imaging sequence with a 720-ms repetition time and 2-mm spatial resolution (isotropic). The images had undergone preliminary preprocessing steps, which included gradient distortion correction, motion correction, distortion correction, normalization to the MNI space, intensity normalization and bias field removal. We employed a multiple regression approach to remove the signal variance over time associated with nuisance variables such as motion parameters, mean white matter signal, cerebrospinal fluid signal and global signal. The residual BOLD time series were band-pass filtered in the frequency range of 0.01 to 0.08 Hz. Any images with frame-wise displacement exceeding 0.5 millimetres were excluded from the analysis. Two datasets had to be excluded due to substantial in-scanner motion, leaving 98 datasets for the analysis. Datasets were smoothed using a Gaussian kernel with a full width at half-maximum of 5 mm, after extracting the unsmoothed time series from the regions of interest described above. Statistical significance was again assessed at cluster level [FWE-corrected *P*-values < 0.05 using *P*(uncorrected) < 0.001 as the cluster defining threshold].

## Results

DaT-SPECT and control groups differed significantly in terms of objective and subjective levels of Parkinsonian motor symptoms as assessed by MDS-UPDRS III, the UES to specifically assess task execution-relevant motor symptoms, and MDS-UPDRS II scores (all *P* < 0.001). In addition, groups differed in terms of symptoms of depression as assessed by beck depression inventory (*P* = 0.010), driven by higher scores in the DaT-SPECT group (5.9 ± 5.2) than in controls (2.9 ± 3.1). However, the mean beck depression inventory group score in the DaT-SPECT participants remained below the beck depression inventory threshold for mild depression. There was no significant difference in Montreal cognitive assessment scores (*P* = 0.073) between the DaT-SPECT group (26.74 ± 2.21) and the control group (27.75 ± 1.86). Within the DaT-SPECT group, rmANOVA with the within-subject factor session (training/retest) revealed no significant differences in terms of overall and specifically task execution-relevant Parkinsonian motor symptoms before the training and retest sessions [MDS-UPDRS III, *F*(1,37) = 0.384; *P* = 0.539; UES; *F*(1,37) = 0.028; *P* = 0.868] excluding systematic contamination of the consolidation assessment by fluctuations in Parkinsonian motor symptoms over the day.

### Behavioural results—general task execution and online/offline learning in the DaT-SPECT and control groups

**Figure 3:**
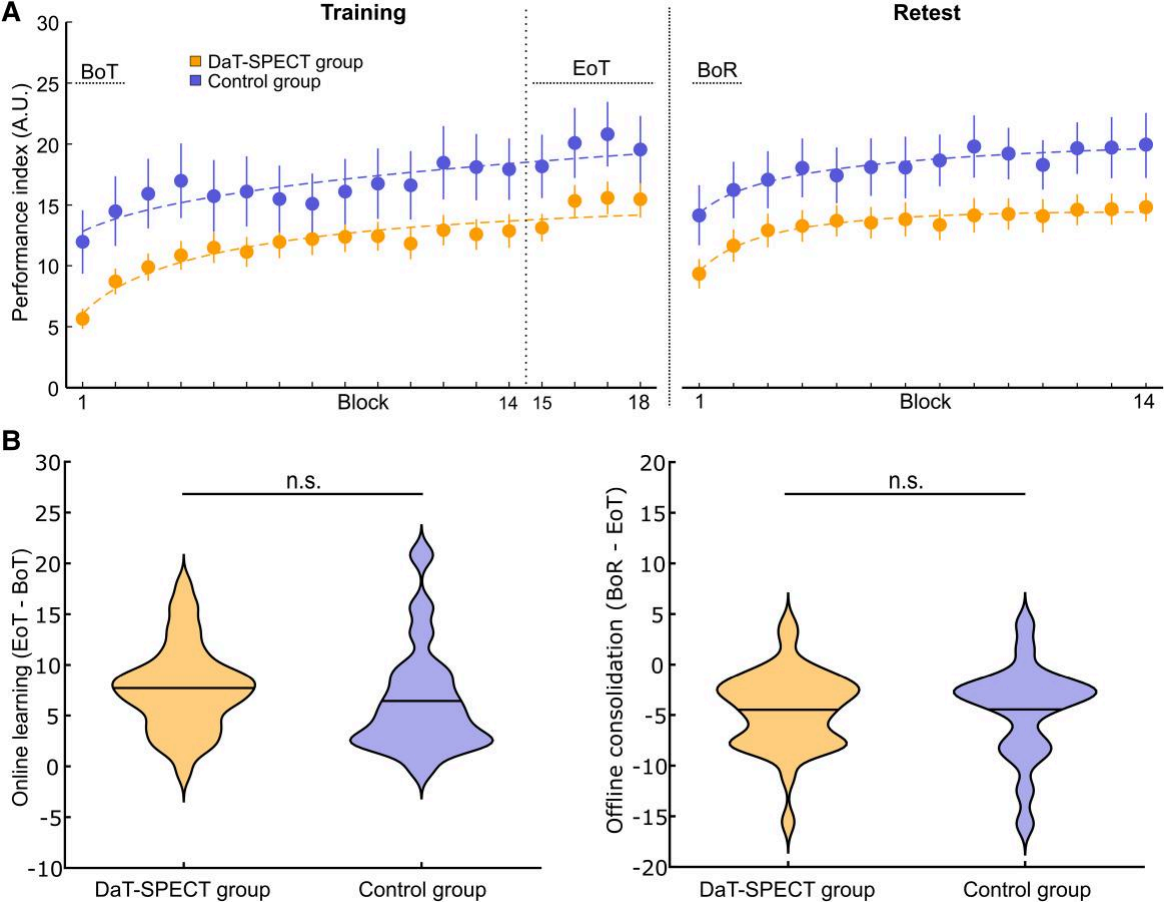
**Motor sequence learning task performance**. **(A**) Training and retest performance of DaT-SPECT (*n* = 38) and control groups (*n* = 20) with important intervals marked (BoT, beginning-of-training performance; EoT, end of training; BoR, beginning of retest. (**B**) Online learning [left; unpaired, two-sided *t*-test, *t*(56) = 1.005; *P* = 0.319] and offline consolidation [right; unpaired, two-sided *t*-test, *t*(56) = 0.061; *P* = 0.952] of both groups (n.s., not significantly different).

In an overall descriptive analysis, we first explored whether our DaT-SPECT group differed at all from age-matched healthy controls in terms of general task performance or online and offline learning. Task performance across the initial training session (i.e. online learning) was compared between groups by applying a mixed rmANOVA to the PI values across blocks of training with the between-subject factor group and the within-subject factor block. This analysis revealed a significant main effect of block [*F*(7.40,414.29) = 33.181; *P* < 0.001] that was driven by increasing PI values across the training session, indicating significant online learning across all participants. However, mean PI across blocks of the training session amounted to 12.02 ± 7.24 in the DaT-SPECT group and to 16.91 ± 12.07 in controls resulting in a trend for the main effect of group [*F*(1,56) = 3.734; *P* = 0.058] pointing to between-group differences in general task performance. Importantly, there was no significant interaction of both factors [*F*(8.787,492.093) = 1.496; *P* = 0.148], indicating that groups did not differ in terms of online learning (Fig. 3A). Accordingly, groups did not differ significantly in terms of the predefined online learning measure (ΔPI EoT—BoT), which averaged 6.4 ± 5.3 in the control group and 7.7 ± 4.2 in the DaT-SPECT group (unpaired, two-sided *t*-test; *P* = 0.319; Fig. 3B). Offline consolidation was assessed by applying a rmANOVA with the within-subject factor time (EoT/BoR) and the between-subject factor group to the mean PI values at EoT and at BoR. RmANOVA revealed a significant main effect of time [*F*(1,56) = 60.846; *P* < 0.001], a trend for the main effect of group [*F*(1,56) = 3.749; *P* = 0.058], but no significant interaction of factors [*F*(1,56) = 0.004; *P* = 0.952], indicating that groups continued to differ in terms of general task performance, but did not relevantly differ in terms of offline motor memory consolidation. Offline consolidation (ΔPI BoR—EoT) amounted to −4.4 ± 3.8 in the DaT-SPECT group, and to −4.5 ± 4.6 in controls, indicating quite similar offline performance losses across the consolidation interval in both groups (Fig. 3B). Of note, rmANOVA conducted on the PI values across blocks of the whole delayed retest session revealed a significant main effect of block [*F*(6.98,390.90) = 13.408; *P* < 0.001], and a significant main effect of group [*F*(1,56) = 4.041; *P* = 0.049] in the absence of a significant interaction of block × group [*F*(6.98,390.90) = 0.391; *P* = 0.907], indicating significant online learning also across the retest session, but again, no significant group difference in the rate of online skill acquisition despite differences in general task performance. Overall, the above results show that online and offline motor sequence learning were relatively intact in the DaT-SPECT group despite higher task execution-relevant Parkinsonian motor symptom load and impaired general task performance compared to age-matched controls.

Of note, resting and/or action tremor were common among participants in the DaT-SPECT group, occurring in 89.5% of cases. However, tremor items from the MDS-UPDRS III were deliberately excluded from the UES score to prevent confounding the analysis of the relationship between striatal DaT availability and task execution-relevant motor symptoms with symptoms that are unrelated to striatal DaT availability (e.g. action tremor in essential tremor) or those that are present at rest but typically subside during task execution (e.g. rest tremor). However, to exclude that action tremor may have significantly influenced task performance and learning dynamics, we conducted an additional analysis comparing task performance and learning dynamics between individuals diagnosed with essential tremor and the control group. The results provided no evidence for a relevant effect of action tremor on motor sequence performance or learning dynamics (Supplementary material).

### Distinct spatial distribution of striatal dopamine depletion associated with Parkinsonian motor symptoms and motor learning impairments

As our primary focus was to examine the effects of striatal dopamine depletion on motor learning, the following analyses focus on the DaT-SPECT group for which data on individual striatal dopamine transporter availability were available.

In a first step, we evaluated whether the individual ability to improve task performance (online and/or offline) or the severity of task execution-relevant Parkinsonian motor symptoms were affected by striatal DaT availability. Correlation analyses revealed that more severe task execution-relevant Parkinsonian motor symptoms (i.e. higher UES values) were significantly associated with lower striatal DaT availability according to SBR values (putamen: *r* = −0.699, *P* < 0.001; caudate nucleus: *r* = −0.619; *P* < 0.001). Similar associations were obtained when using the local SBR in the DaTStriatum volume (with average local SBR intensity: *r* = −0.698, *P* < 0.001; with maximum local SBR value: *r* = −0.729, *P* < 0.001). The medio-lateral location (MNI × coordinate, lower numbers indicate a more lateral location) of the maximum intensity voxel within the DaTStriatum volume was also significantly correlated with the UES (*r* = 0.557, *P* < 0.001; Supplementary Fig. S1A). This was the case for the centre of gravity of all DaTStriatum voxels as well (*r* = 0.479, *P* = 0.002, Supplementary Fig. S1B). Thus, participants with higher UES scores—indicating more severe motor impairment relevant to task execution—displayed relatively higher voxel intensities in more medial sub-parts of the striatum. This pattern aligns with a more pronounced dopamine loss in the lateral putaminal areas of the striatum among individuals with greater motor symptom severity. Of note, we observed no significant correlation of online learning with caudate nucleus and putamen SBR values according to the Hermes BRASS tool (putamen: *r* = 0.192, *P* = 0.249; caudate nucleus: *r* = 0.131, *P* = 0.435) and with local SBR in the DaTStriatum volume (*r* = 0.134, *P* = 0.422). We found only a weak association between offline motor memory consolidation and striatal Hermes BRASS SBR values (putamen: *r* = 0.344, *P* = 0.034; caudate nucleus: *r* = 0.390, *P* = 0.015, Supplementary Fig. S2A and S2B). However, visual inspection of the scatter plots of the latter correlation were suggestive of an outlier driven effect which was, in addition, not reproduced when applying the local SBR approach (*r* = 0.253, *P* = 0.125, Supplementary Fig. S2C).

**Figure 4:**
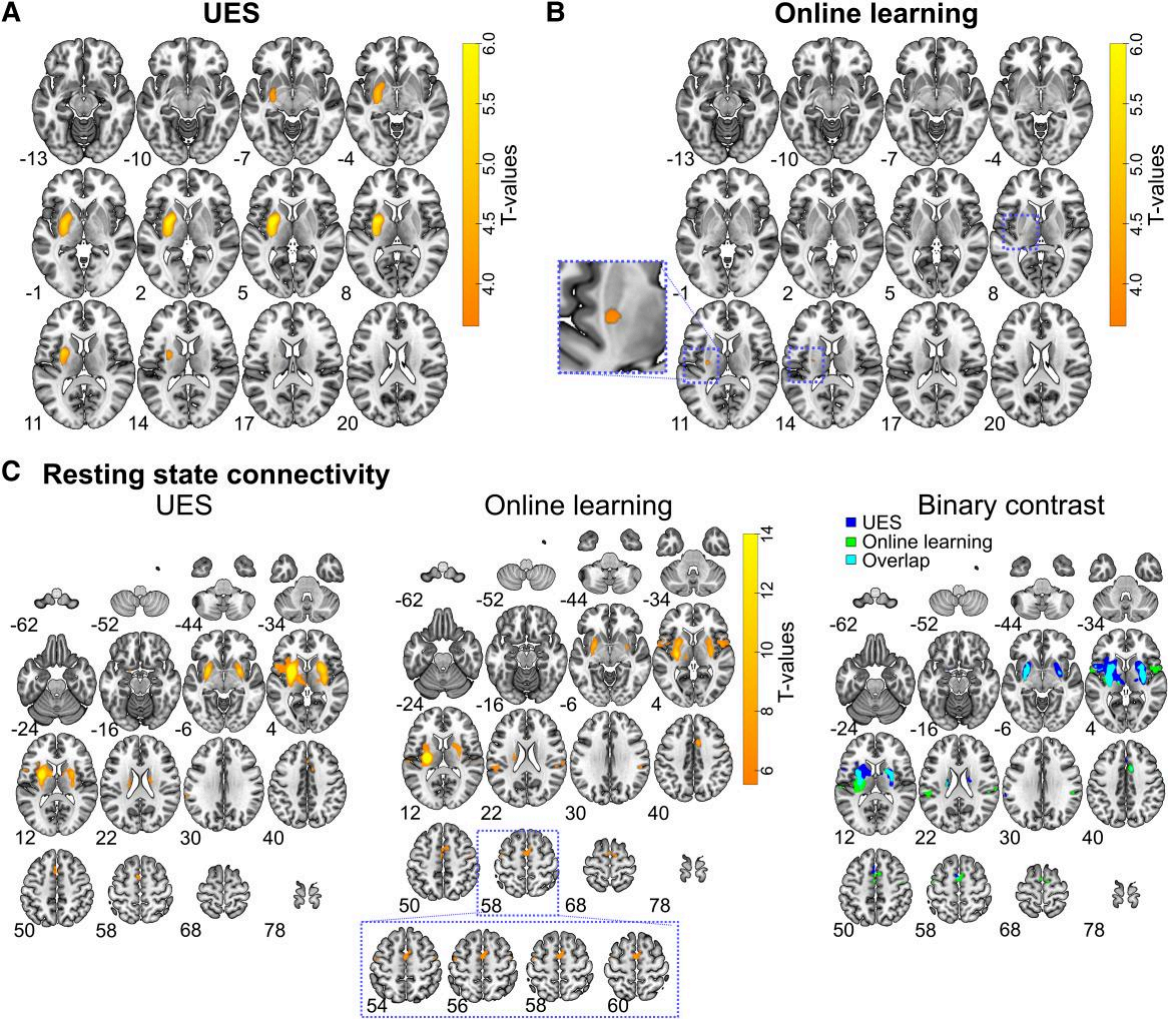
**Voxelwise regression and normative resting state connectivity: local SBR voxels within the DaTStriatum volume significantly associated with UES (A) and online learning (B).** Significant voxels are coloured (*n* = 38, *t*-test FWE-corrected at cluster-level; *P* < 0.05). The significant cluster for online learning is highlighted with a dotted square (in slices corresponding to *z*-coordinate 8, 11 and 14) and the largest cluster (at *z*-coordinate 11) is depicted at the inset on the left side of the panel. (**C**) Voxels exhibiting significant resting state connectivity to the striatal ROI set around the peak voxel associated with UES (far left) and online learning (middle; in both panels computed from 100 healthy persons from the human connectome project as detailed in the ‘Materials and methods’ section, *t*-test FWE-corrected at cluster level *P* < 0.05). On the far right, the two maps (UES & Online learning) are overlaid (Overlap) with a binary contrast. Note the more posterior connectivity to the supplementary motor area and more M1 involvement for online learning compared to UES. Substantial overlap existed in particular at the level of the basal ganglia. Numbers indicate the MNI *z*-coordinate of the slices.

To assess whether striatal DaT availability in specific parts of the striatum was associated with Parkinsonian motor symptom load and online learning/offline consolidation, we computed voxelwise regressions of behavioural data and local SBR intensity distributions within the DaTStriatum volume as detailed in the methods section. As expected, we found a large significant cluster in the DaTStriatum volume, spanning most of the striatum, in which DaT-SPECT signal intensity was associated with contralateral Parkinsonian motor symptom severity [UES, Fig. 4A, *t*-test FWE-corrected at cluster level: *P* < 0.001; peak voxel at MNI coordinate (−28;0;6), *t*-test FWE-corrected: *t* = 6.77; *P* < 0.001]. For online motor learning, we found a smaller significant cluster in the more posterior and dorsal regions of the striatum, which partially overlapped with the cluster related to the UES [Fig. 4B;  *t*-test FWE-corrected at cluster level: *P* = 0.027; peak voxel at MNI coordinate (−28;−16;12), *t*-test FWE-corrected: *t* = 4.00, *P* = 0.019]. No significant cluster was found for offline consolidation of training-induced performance improvements.

We then used the peak intensity voxels within the significant clusters as seeds for a normative resting-state connectivity analysis (*t*-test FWE-corrected at cluster level; *P* < 0.05). For the UES-associated seed, we found significant connectivity predominantly to the supplementary motor area and basal ganglia (Fig. 4C, left panel). When using the online learning peak intensity voxel as seed, a similar pattern of connectivity emerged with, however, stronger and more posterior involvement of the supplementary motor area and, importantly, the ipsilateral primary motor cortex (Fig. 4C, middle and right panel). Overall, these results indicate a distinct spatial distribution of striatal dopamine-dependent function and connectivity associated with online motor learning and Parkinsonian motor symptom severity, but not offline motor memory consolidation.

### Impaired online learning in participants showing striatal dopamine depletion suggestive of a neurodegenerative Parkinsonian disorder

**Figure 5:**
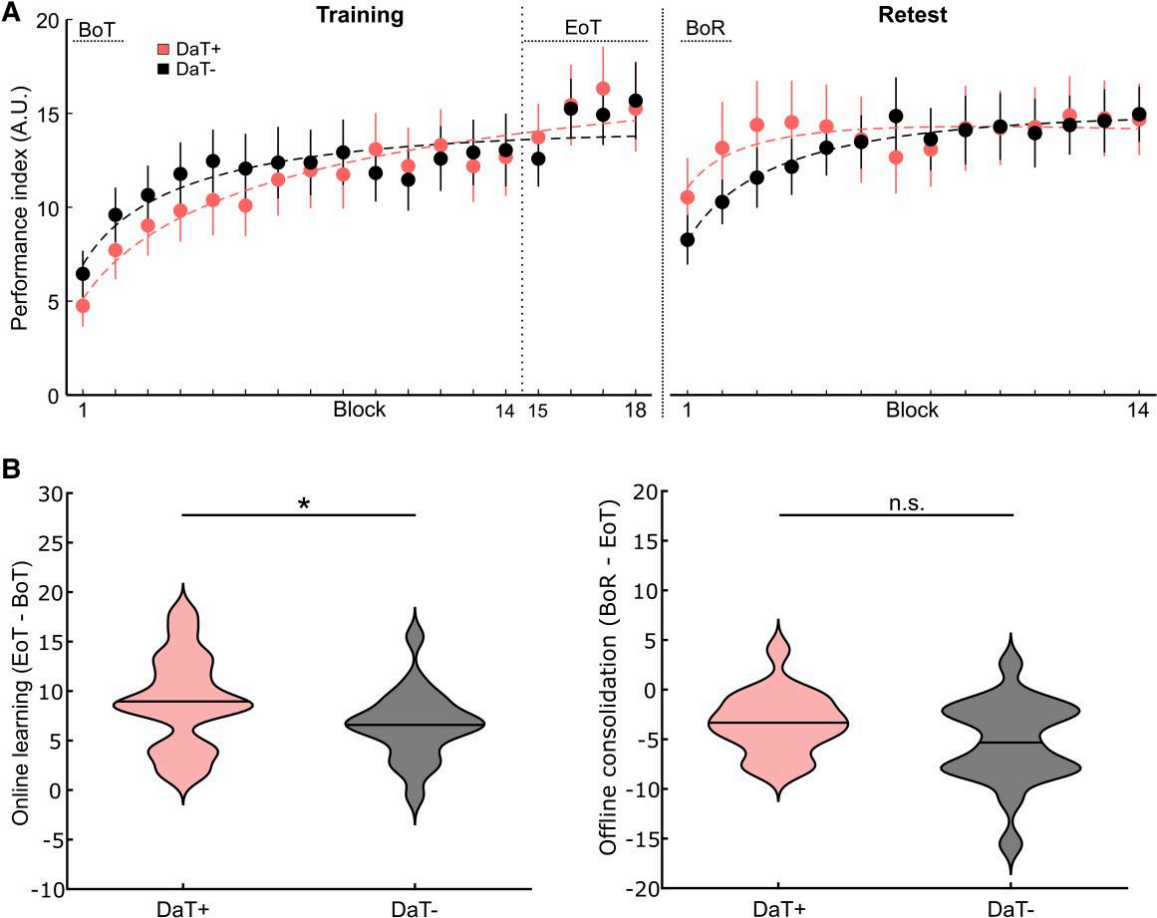
**Online learning and offline consolidation in individuals with normal striatal dopamine transporter availability (DaT+) and impaired striatal dopamine transporter availability (DaT–)**. (**A**) Training and retest performance of DaT+ (*n* = 18) and DaT− (*n* = 20) sub-groups. (**B**) Online learning (left) and offline consolidation (right) of both sub-groups. We found a significant difference for online learning with significantly greater increase in performance in the DaT+ sub-group [unpaired one-sided *t*-test, *t*(36) = 1.785; *P* = 0.041].

Based on the above results, we hypothesized that participants with abnormal striatal DaT depletion suggestive of Parkinson’s disease, according to cut-offs used for clinical purposes, should show impairments in online motor learning compared to participants with ‘normal’ striatal DaT availability. We, therefore, evaluated in an additional exploratory analysis, whether online and offline motor learning differed within the DaT-SPECT group when dichotomized into cohorts of participants with evidence of striatal DaT depletion suggestive of a neurodegenerative Parkinsonian disorder according to age-adjusted putaminal *z*-scores (according to the Hermes BRASS tool) of <−2.5 (DaT−, *n* = 20, 5 female; mean age, 63.40 ± 7.80) and participants with putaminal *z*-scores of ≥−2.5 (DaT+, *n* = 18, 9 female; mean age, 66.2 ± 8.5). As anticipated, the DaT-group exhibited more pronounced Parkinsonian motor symptoms relevant to task execution, reflected by significantly higher UES scores compared with the DaT+ group (mean UES, DaT+ group: 1.67 ± 2.14, DaT− group: 7.20 ± 2.86; Mann–Whitney U-test; *P* < 0.001). RmANOVA conducted on the PI values across blocks of training with the between-subject factor group (DaT+/DaT−) and the within-subject factor block did not reveal a significant main effect of group [*F*(1,36) = 0.052; *P* = 0.820], indicating that groups did not relevantly differ in terms of general task performance. However, rmANOVA revealed a significant main effect of block [*F*(6.91,248.86) = 32.562; *P* < 0.001] and a significant interaction of block × group [*F*(6.91,248.86) = 2.043; *P* = 0.035] indicating differences in the rate of online learning between groups (Fig. 5A). This effect was driven by larger mean PI improvement across the training session (ΔPI EoT—BoT) of 9.0 ± 4.7 in the DaT+ group compared with 6.6 ± 3.5 in the DaT− group (one-sided, unpaired *t*-test; *P* = 0.041; Fig. 5B). Offline consolidation was assessed by applying a rmANOVA with the within-subject factor time and the between-subject factor group to the mean PI values at EoT and BoR. RmANOVA revealed a significant main effect of time (EoT/BoR) [*F*(1,36) = 51.215; *P* < 0.001] that was driven by offline performance losses across the consolidation interval in both groups (PI ΔBoR—EoT, DaT−: −5.3 ± 4.2; DaT+: −3.3 ± 3.1; Fig. 5B). However, the rmANOVA showed that there was no significant main effect of group [*F*(1,36) = 0.404; *P* = 0.529], and no significant interaction of factors [*F*(1,36) = 2.720; *P* = 0.108], indicating that the groups did not significantly differ in terms of offline motor memory consolidation or general task performance. In conclusion, this additional exploratory analysis indicates that DaT-SPECT evidence of putaminal dopamine depletion suggestive of a neurodegenerative Parkinsonian disorder is associated with impairments in the ability to acquire sequential motor skills online despite relatively similar general task performance in the DaT+ and DaT− groups.

## Discussion

In this study, we explored how the availability and distribution of striatal dopamine transporters affects the acquisition of a new dexterous motor skill in individuals with and without evidence of striatal dopamine depletion according to DaT-SPECT. While we observed no significant association between motor sequence learning and striatal DaT availability when averaged across the entire striatal volume, we did uncover distinct spatial patterns of striatal DaT reduction that were linked to difficulties in online motor skill acquisition and Parkinsonian motor symptoms, respectively. Specifically, whereas significant associations between striatal DaT reduction and task execution–relevant Parkinsonian motor symptoms showed a widely distributed pattern across the contralateral striatum with an anterior maximum, significant associations between striatal DaT depletion and impairments in online motor learning were found in circumscribed posterior putaminal parts of the striatum.

In humans, functional neuroimaging studies provided substantial evidence that the striatum serves as a key node in the motor learning network, with its associative and sensorimotor sub-domains being dynamically engaged throughout the learning process.^13,55,56^ While the caudate nucleus, which is linked to the associative striatum, is involved in the very early rapid learning phase, more advanced learning phases, during which performance improves more slowly and gradually, are associated with increasing recruitment of the putamen, which accounts for a large part of the sensorimotor striatal sub-domain.^57,58^ Throughout the learning process, dynamic shifts in activation are also evident within the putamen itself, transitioning from the anterior associative to the posterior sensorimotor putaminal sub-regions with repeated practice.^13^ Of note, such increases in the extent of posterior putamen recruitment associated with ‘later’ learning phases were observed even after only 10 min of practice of a simple motor task similar to the task applied here.^13^ Causal evidence supporting the crucial role of the striatum in motor learning primarily originates from animal studies, which have shown impaired motor skill acquisition following striatal lesions.^59,60^ In rodents, the reorganization of motor learning–related cellular plasticity within the sensorimotor striatum has been shown to be mediated by finely tuned DaT-regulated dopamine release.^21^ In humans, evidence supporting the central role of the striatum in motor sequence learning was recently provided by Wessel *et al*.,^61^ who demonstrated that targeting the striatum by transcranial temporal interference stimulation led to increased putaminal activity and enhanced motor sequence learning. Given the compelling evidence for the central role of the striatum in motor learning, our findings showing a specific association between DaT depletion in the posterior sensorimotor striatum and impaired online motor sequence learning suggest that nigro-striatal dopaminergic neurotransmission directed to sensorimotor parts of the putamen represents a key mechanism mediating training-induced acquisition of motor skills.

Our normative resting-state functional connectivity analyses based on striatal voxels that showed maximum association with online motor learning and task execution–relevant Parkinsonian motor symptoms revealed quite similar overlapping patterns for both behavioural domains, mainly involving significant connectivity within the basal ganglia and with the supplementary motor area. However, only the connectivity pattern specifically associated with striatal DaT-depletion-related modulation of online motor learning also encompassed significant connectivity with primary motor cortical areas (M1), in addition to dorsal predominance of functional connectivity with the supplementary motor area. This is well compatible with the ample evidence showing that M1 has, besides its key role in motor execution, a pivotal role in motor learning^1,3,8,55,62,63^ and was discussed in this context as a ‘tutor’ for downstream sub-cortical motor circuit plasticity.^64^ Moreover, we know from animal studies in rodents that the dorsolateral (i.e. putaminal/sensorimotor) striatum is a major target for M1 output neurons, which are recruited during motor learning.^65^ It was further demonstrated that the outputs of these M1 engram neurons also undergo learning-related synaptic plasticity, as their projections to the sensorimotor striatum are strengthened with ongoing practice.^65^ Of note, our finding of significant motor learning-related functional connectivity between M1 and the posterior parts of the putamen matches well with studies in humans showing that M1 is also structurally connected to specifically posterior (sensorimotor) parts of the putamen.^66-68^ Motor memory traces in M1 could therefore propagate along these connections and control the induction of learning-related plasticity in the sensorimotor striatum that supports the acquisition of motor skills. The significant association of impaired online motor sequence learning with DaT depletion specifically in posterior putaminal areas demonstrated here may, thus, indicate that learning-related M1-driven plasticity induction in the sensorimotor striatum is dopamine-dependent.

One might, therefore, expect motor sequence learning to be significantly impaired under conditions of putaminal dopamine depletion beyond a certain threshold. In addition to significant differences regarding task execution–relevant Parkinsonian motor symptoms, we also found evidence of deficits in online acquisition of sequential motor skills at the group level in individuals with a reduction of putaminal DaT availability indicative of a neurodegenerative Parkinsonian disorder compared with participants with ‘normal’ age-adjusted putaminal DaT availability.

By far, the largest portion of knowledge about potential effects of striatal dopamine depletion on motor learning in humans originates from studies that investigated motor learning in Parkinson’s disease.^22,27^ However, the results of previous studies, which predominantly investigated early online motor skill acquisition, have been inconsistent with respect to the question whether Parkinson’s disease is associated with deficits in motor skill acquisition.^14,22,23,25,27-30,37,69,70^ Our current findings linking putaminal dopamine depletion to impairments of online motor skill acquisition further support the majority of the above studies suggesting an impairment of online motor sequence leaning in Parkinson’s disease.^23,26,28,30,69^ Although other neurotransmitter systems are also frequently affected in Parkinson’s disease, our study suggests a direct link between impaired motor skill acquisition and striatal dopamine deficiency. Studies showing progressively impaired learning with ongoing disease progression may further support this conclusion.^24,71^ In terms of effects of Parkinson’s disease on offline motor sequence memory consolidation, existing evidence suggests that consolidation is relatively intact in Parkinson’s disease, both across periods of rest over the day and over the night.^28,31,32^ Our current findings, which indicate no significant relationship between striatal dopamine depletion and motor memory consolidation, align with this research. This would suggest that the offline motor memory consolidation phase may be less dependent on striatal dopaminergic mechanisms than the online learning phase. Nevertheless, although not statistically significant, consolidation appeared to be reduced in individuals with pronounced putaminal DaT depletion. Moreover, our participants diagnosed with Parkinson’s disease were, by design, in a rather early disease stage; hence, we cannot dismiss the possibility that deficits in motor memory consolidation might emerge in later disease stages as nigro-striatal degeneration progresses. In summary, our study provides novel evidence supporting the hypothesis that deficits in motor learning in Parkinson’s disease may be causally linked to striatal dopamine depletion, and at least partly independent of Parkinsonian motor execution impairments due to striatal dopamine depletion.

Against this background, the question arises whether motor learning deficits in Parkinson’s disease may be specifically relieved by pharmacological interventions that compensate for nigro-striatal dopamine depletion independent of transient effects on Parkinsonian motor symptoms? This is highly relevant, as the ultimate goal of interventions to promote motor learning is not to induce temporary changes in motor performance but to induce lasting improvements in skill performance. Interestingly, a recent study indeed demonstrated that long duration response to levodopa in Parkinson’s disease (i.e. persisting improvements of motor function up to days after treatment discontinuation independent of peripheral levodopa pharmacokinetics^72^) may synergistically interact with motor learning to induce lasting adaptive changes in neuroplasticity in cortico-sub-cortical networks.^73^ Although not addressed in our study, it seems plausible that also dopamine-dependent processes, such as reward-based learning, may contribute to the synergistic effects of training and levodopa on long-term motor memory formation in Parkinson's disease.^74,75^ Further evidence that targeting (in this case aging-related) striatal dopamine depletion by dopaminergic medication may synergistically improve motor learning is provided by studies in healthy older individuals and individuals with stroke in whom levodopa facilitated motor learning^33-35,76^

However, although there is some evidence that levodopa may also improve motor learning in Parkinson’s disease^36^ others observed no or even detrimental effects of levodopa on motor skill acquisition^37,39,40^ even when controlling for an optimal level of dopaminergic stimulation to alleviate Parkinson’s disease motor symptoms.^38^ This may be explained by the dopamine-overdose hypothesis proposing that dopaminergic medication may restore dopamine levels in depleted parts of the brain (i.e. in the sensorimotor striatum), but overdose brain regions in which dopamine depletion is less pronounced (i.e. associative striatum) and interfere with normal function in these areas.^39,40,77,78^ Overall, the evidence available suggests that motor learning may be promoted by dopaminergic medication in Parkinson’s disease, but different individual levels of dopaminergic stimulation may be necessary to improve Parkinsonian motor symptoms and facilitate motor learning, respectively.

## Conclusion

Our study demonstrated that the spatial distribution of striatal DaT availability associated with the expression of Parkinsonian motor symptoms differs from the spatial pattern associated with motor learning. This indicates that motor sequence learning is affected by striatal dopamine depletion independent of motor execution impairments due to Parkinsonian motor symptoms. Specifically, we observed that impaired motor skill acquisition is related to dopamine depletion in posterior sensorimotor areas of the putamen, which are connected to the primary motor cortex. This may indicate that specifically motor learning–related recruitment of sensorimotor parts of the putamen and cortico-striatal plasticity is dopamine dependent. Future studies are needed to address whether and how dopamine substitution therapy may be utilized to facilitate motor learning in Parkinson’s disease and promote long duration learning effects that persist beyond immediate effects on mere motor execution.

## Supplementary Material

fcae409_Supplementary_Data

## Data Availability

Data privacy statements signed by all subjects protect personal data. The data can be made available upon specific request taking into account the opinion of the local data privacy board. The Matlab code used in this publication is made available at: https://github.com/ChrisF48/MotorLearningPutamen.
