# Supplementary material for: Motor learning is modulated by dopamine availability in the sensorimotor putamen
Source: Brain Commun. 2024 Nov 13;6(6):fcae409. doi: 10.1093/braincomms/fcae409 (PMC11582004; doi:10.1093/braincomms/fcae409)
Supplement: fcae409_Supplementary_Data [file fcae409_supplementary_data.docx]

**Supplementary Material**

**Potential influence of action tremor on task performance and learning dynamics**

To exclude that action tremor may have significantly influenced task performance and learning dynamics, we conducted an additional analysis comparing task performance and learning dynamics between individuals diagnosed with essential tremor (exhibiting action tremor) and the control group. To this end, we computed a repeated measures ANOVA on the performance index values across blocks of the initial training session with the between-subject factor Group (individuals diagnosed with essential tremor/healthy controls) and the within-subject factor Block. This analysis revealed a significant main effect of Block (F_(6.43,561.61)_=26.158, p<0.001), but no significant main effect of Group (F_(1,34)_=2.626, p=0.114) nor a significant interaction of both factors (F_(6.43,37.19)_=1.732, p=0.109). Therefore, the results provide no evidence for a relevant effect of action tremor on motor sequence performance or learning dynamics compared to healthy controls.

**Supplementary Figures**


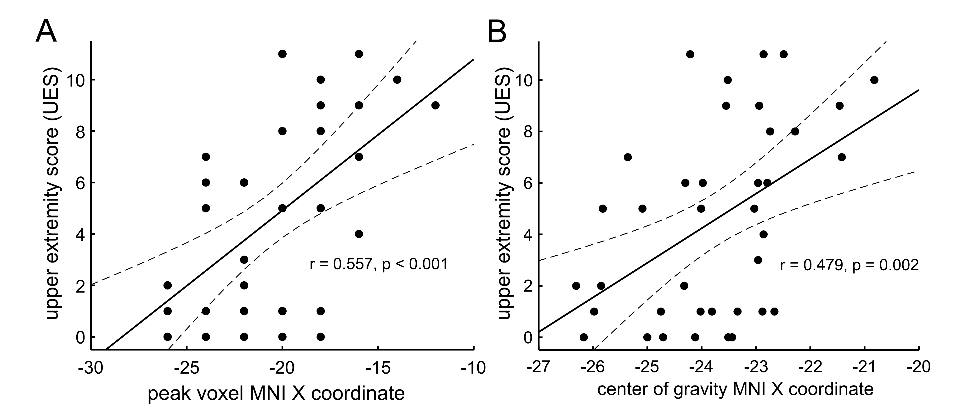


**Supplementary Figure 1:** Correlations of the peak voxel MNI X coordinate (A) and the center of gravity MNI X coordinate (B) with Parkinsonian motor symptom severity as represented by the upper extremity score (UES). Higher intensity voxels were associated with more medial dopamine availability in participants with more severe motor impairment suggesting a loss of dopamine availability predominantly in more lateral parts of the striatum.


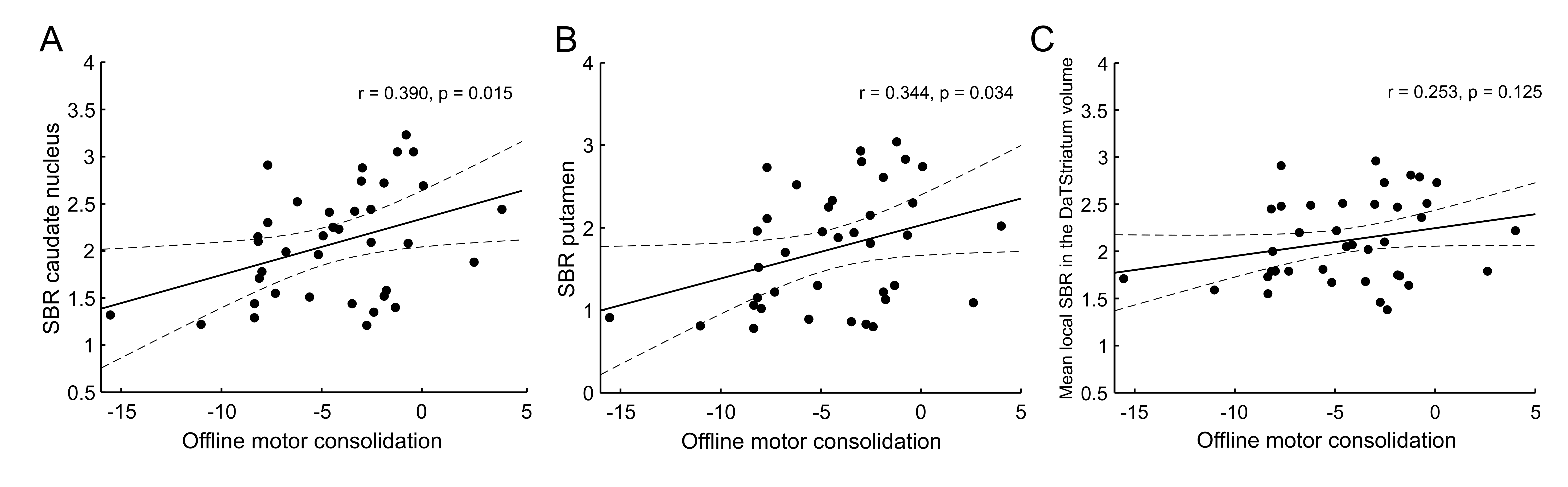


**Supplementary Figure 2:** Correlations of offline motor consolidation and dopamine availability according to BRASS (caudate: A, putamen: B) as well as the DaTStriatum local SBR approach (C). The correlations in panels A and B were significant, though they seemed to be largely influenced by outliers (significances lost when removing the leftmost and rightmost data point).
